# Experiences and preferences towards collecting a urine and cervicovaginal self-sample among women attending a colposcopy clinic

**DOI:** 10.1016/j.pmedr.2022.101749

**Published:** 2022-02-28

**Authors:** Mirte Schaafsma, Rianne van den Helder, Maaike C.G. Bleeker, Fleur Rosier-van Dunné, Irene A.M. van der Avoort, Renske D.M. Steenbergen, Nienke E. van Trommel

**Affiliations:** a Antoni van Leeuwenhoek/Netherlands Cancer Institute, Department of Gynecologic Oncology, Center of Gynecologic Oncology Amsterdam, Amsterdam, the Netherlands; b Amsterdam University Medical Centers, Department of Pathology, Cancer Center Amsterdam, Amsterdam, the Netherlands; c Tergooi Medical Center, Department of Obstetrics and Gynecology, Blaricum, the Netherlands; d Ikazia Hospital, Department of Obstetrics and Gynecology, Rotterdam, the Netherlands

**Keywords:** Urine collection, Self-sampling, Experience, Preference, Cervical cancer

## Abstract

The effectiveness of cervical cancer screening is hampered by low attendance rates. The collection of a urine sample is hypothesized to engage non-attenders in cervical cancer screening. The aim of this prospective cohort study was to evaluate experiences of women on urine collection and cervicovaginal self-sampling in a home-based setting and preferences for future cervical cancer screening. This study included 140 women, with a median age of 40 years, who were planned for a large loop excision of the transformation zone (LLETZ) procedure. All women collected a urine sample using conventional urine cups and a cervicovaginal self-sample prior to the LLETZ in a home-based setting. Following sample collection, women filled in a questionnaire. Results showed that the instructions of urine collection and cervicovaginal self-sampling were considered clear (95%, 95%CI: 88–98; 92%, 95%CI: 83–96, respectively). Women considered urine collection compared to cervicovaginal self-sampling to be more acceptable (*p* < 0.001), and to provide more reliable results (*p* < 0.001). The three highest reported preferred sampling methods for future cervical cancer screening were: urine collection (n = 39, 28%, 95%CI: 19–39), clinician-taken cervical scrape (n = 32, 23%, 95%CI: 15–34), and equal preference for urine collection, clinician-taken cervical scrape and cervicovaginal self-sampling (n = 30, 21%, 95%CI: 14–32). In conclusion, urine collection and cervicovaginal self-sampling are acceptable sampling methods, considered easy to collect in a home-based setting, and moreover, considered trustworthy. Although these results are promising, more research is required to determine if urine collection also lowers the barrier for non-attendees and, thereby, increases the attendance rates of cervical cancer screening.

## Highlights

• In a colposcopy referral population, the majority of women consider self-collection of a urine sample and a cervicovaginal self-sample acceptable and easy to collect in a home-based setting.
• Urine collection is worth investigating as a potential screening method to possibly improve attendance rates in cervical cancer screening.

## 1 Introduction

The effectiveness of cervical cancer screening largely relies on its attendance rates (Aitken et al., 2021). In the Netherlands only 50% to 58% of the invited women aged between 30 years and 60 years, actually attended cervical cancer screening in 2017 to 2020 (Integraal Kankercentrum Nederland, 2021). This is worrisome as studies have demonstrated that non-attenders are more frequently diagnosed with advanced stages of cervical cancer and have an increased risk for cervical cancer-related mortality (Bchtawi et al., 2019, Jansen et al., 2020). Especially invited women with lower socio-economic status, a migration background, and unmarried and solo-living women attend less often cervical cancer screening (Aitken et al., 2021). Factors that may affect screening-attendance are organizational barriers (e.g. forgetting to schedule an appointment), practical barriers (e.g. a cervical scrape been taken in another context over the last three years, being pregnant, having fertility treatment, or breastfeeding), and psychological barriers (e.g. anxiety and embarrassment for having a cervical scrape taken) (Bosgraaf et al., 2014).

To improve attendance rates, it is important to remove barriers that may decrease screening-attendance. Offering the option of attending the cervical cancer screening through home-based collection of a cervicovaginal self-sample or a urine sample, either using conventional methods or a first void collection device, could overcome some of these barriers. This may consequently lead to an increase in attendance and effectiveness of cervical cancer screening (Tranberg et al., 2020). Especially urine collection looks promising as it has been reported as the most preferred sampling method for cervical cancer screening in several studies (Leeman et al., 2017, Ørnskov et al., 2021, Rohner et al., 2020, Sargent et al., 2019, Tranberg et al., 2020). However, urine collection methods differ in these studies and few studies have compared the collection of complete urine void without special devices to cervicovaginal self-sampling in a home-based setting (Leeman et al., 2017, Ørnskov et al., 2021, Rohner et al., 2020, Sargent et al., 2019, Tranberg et al., 2020).

Therefore, the aim of this study was to investigate the experiences and preferences of home-based collection of a complete urine void compared to cervicovaginal self-sampling among women referred for colposcopy.

## 2 Methods

### 2.1 Study population

The questionnaires were collected from women participating in the SOLUTION 2 study. The SOLUTION 2 study is a prospective cohort study, which aimed to determine the performance of high-risk human papillomavirus (hrHPV) DNA and DNA methylation markers to detect high-grade cervical intraepithelial neoplasia (CIN) in urine.

At two colposcopy clinics Dutch-speaking women aged 18 years and above, diagnosed with a high-grade CIN lesion (CIN2 or CIN3) and planned for a LLETZ procedure after colposcopy were asked to participate in this study. The instructions of the sampling collection were briefly explained by a physician. Women were instructed to collect two samples in a home-based setting in the days before the LLETZ procedure: a complete urine void irrespective of time of collection and personal hygiene, and secondly a cervicovaginal self-sample (dry brush device, Evalyn® Brush, Rovers Medical Devices, Oss, The Netherlands). The women who were willing to participate, received a package with patient information forms, consent forms, sampling kits and a questionnaire. Furthermore, each patient received a written instruction for urine collection and a picture-based and written instruction for cervicovaginal self-sample collection. For urine collection patients were asked to decant free-catch urine void into three 30 mL collection cups containing 2 mL 0.6 M Ethylenediaminetetraacetic (EDTA) to preserve the DNA quality. Following sample collection, women were asked to fill in the questionnaire by themselves. Subsequently, they mailed back the samples, the questionnaire, and the written informed consent to the laboratory of the Amsterdam University Medical Centers. All women provided written informed consent. After arrival in the laboratory, samples were tested for hrHPV DNA and DNA methylation markers, of which the results will be reported elsewhere (Van den Helder et al., submitted).

Ethical approval for the SOLUTION 2 study was provided by the Medical Ethical Committee of the VU University Medical Centre (no 2017.112).

### 2.2 Questionnaire

The questionnaire used was based on a previous study reporting on patient experiences regarding cervicovaginal self-sampling (Polman et al., 2019). It consisted of twelve questions divided into four categories: patient characteristics and cervical cancer screening history, experiences with clinician-taken cervical scrapes, experiences with urine collection and cervicovaginal self-sampling, and the participant’s sampling preference for future cervical cancer screening. Responses regarding the experiences were gathered with a 5-point Likert scale ranging from 1, the most negative result, to 5, the most positive result. The original Dutch questionnaire was translated to English and added as Supplement 1.

### 2.3 Statistical analysis

General characteristics were described by median and interquartile range (IQR) for continuous variables and counts with percentages and confidence intervals (CI) for categorical variables. Likert plots were used to visualize the responses on questions. The Wilcoxon signed ranks test was performed to analyze the differences between the women’s experiences of collecting a urine sample and a cervicovaginal self-sample. In the results section, women’s experiences are presented by the cumulative percentages of the positive and extremely positive responses. For every question the responses of the 5-point Likert scale were compared between the experiences for urine collection versus the experiences of cervicovaginal self-sampling by the paired Wilcoxon signed ranked test analysis. The Chi square test was performed to analyze differences in the sampling preference between women who were diagnosed by cervical cancer screening and women who were referred because of complaints. All data analyses were performed in R version 4.0.3.

## 3 Results

### 3.1 Study population

In total, 144 women participated in the SOLUTION 2 study, of whom 140 (97%) filled in the questionnaire. The general characteristics of all included women (n = 140) are indicated in Table 1. Data were partially missing in 19 women (14%). When we compared the women who skipped one or more questions (n = 19) to the women who completed the questionnaire (n = 121) no differences were seen in the screening history (p = 0.907) or educational level (p = 0.639). However, the women who skipped one or more questions were significantly older (median age of 45; IQR: 37.5–50.5) compared to the women who completed the questionnaire (median age 38; IQR: 31.0–45.0) (p = 0.031). All responses were included in the analysis.

**Table 1:** General characteristics of the study population (n = 140). Abbreviations: IQR = interquartile range.

**Age in years (median (IQR)):**	40 (31–46)
	
**Age**	**n (%)**
20–29 years	12 (9)
30–39 years	56 (40)
40–49 years	42 (30)
50–59 years	26 (19)
60–69 years	4 (3)

**Education**	**n (%)**
No or primary school	1 (1)
Secondary education	29 (21)
Secondary vocational education	45 (32)
Higher professional education	51 (36)
University	12 (9)
Missing	2 (1)

**History of cervical samples taken**	**n (%)**
History of attending cervical cancer screening	104 (74)
- History of cervical cancer screening only	85 (61)
- History of both cervical cancer screening and opportunistic screening	19 (14)
No history of attending cervical cancer screening	36 (26)
- No history of cervical cancer screening or opportunistic screening	1 (1)
- No history of cervical cancer screening but a history of opportunistic screening.	35 (25)

**Interval between last cytology and diagnosis**	**n (%)**
0–6 years	132 (94)
7–12 years	5 (4)
Unknown	3 (2)

**Reason of cervical cytology collection**	**n (%)**
Cervical cancer screening	94 (67)
- Cervical scrape taken by a general practitioner	86 (61)
- Cervicovaginal self-sampling	8 (6)
Complaints	33 (24)
Other	13 (9)

### 3.2 Patients’ characteristics and cervical cancer screening history

The median age of the included women was 40 (range: 22–69, IQR: 31–46, Table 1). The majority of the women completed secondary vocational (n = 45, 32%) or higher professional education (n = 51, 36%). A total of 104 women (74%) had a history of attending cervical cancer screening at least once. The majority of women were referred for colposcopy because of an abnormal result of the cervical cancer screening (n = 94, 67%), of whom eight women (6%) attended through cervicovaginal self-sampling. In total 33 women (24%) were referred because of complaints. Other women (n = 13, 9%) were screened at their own initiative (n = 11, 8%) or because of unknown reasons (n = 2, 1%).

### 3.3 Experiences with clinician-taken cervical scrapes

The experiences with clinician-taken samples are described in Supplemental Table 1. The following percentages and CIs are calculated after excluding the missing responses.

Most of the responding women (n = 104, 75%; 95% CI: 64–83) felt somewhat comfortable or comfortable when the cervical scrape was taken by the clinician and the majority (n = 78, 57%; 95% CI: 45–67) experienced the sampling as a little painful or not painful at all. Almost all responding women (n = 124; 91%; 95% CI: 82–95) were confident or extremely confident of correct collection of the clinician-taken cervical scrape.

### 3.4 Experiences with urine collection and cervicovaginal self-sampling

**Fig. 1:**
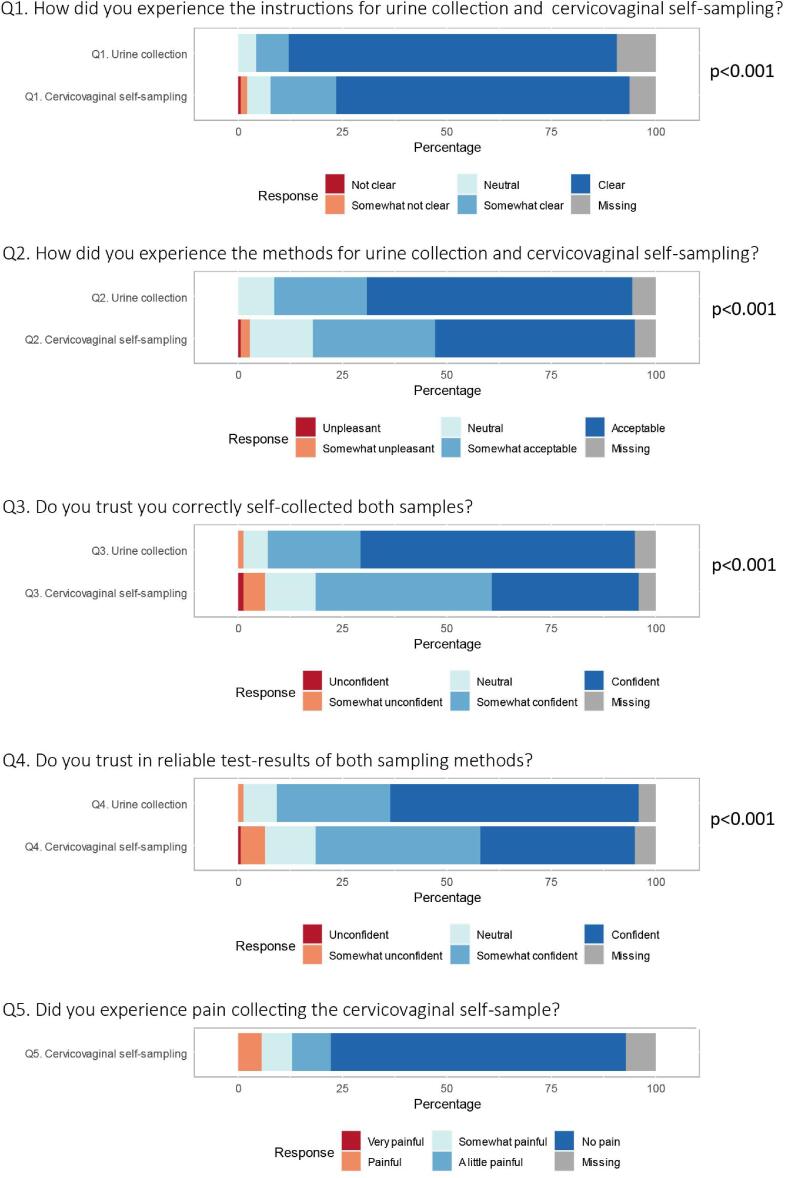
Experiences of urine collection and cervicovaginal self-sampling (n = 140). Missing responses are presented in grey on the right side.

The experiences with urine collection and cervicovaginal self-sampling are summarized in Fig. 1. The results are detailed in Supplemental Table 1. In contrast to Supplemental Table 1, missing responses of women are excluded from the reported percentages and CIs in the following paragraph.

Instructions for sample collection using both methods were experienced as somewhat clear or clear: 121 (95%; 95% CI: 88–98) women experienced the instructions for urine collection as clear versus 120 (92%; 95% CI: 83–96) for cervicovaginal self-sampling. The majority of women experienced both methods of sampling as somewhat acceptable or acceptable: 120 (91%; 95% CI: 82–96) for urine collection versus 108 (81%; 95% CI: 71–89) for cervicovaginal self-sampling. In comparison, urine collection was considered as more acceptable than cervicovaginal self-sampling (*p* < 0.001). Considering the sampling method, the majority of women were somewhat confident or confident that they correctly collected the urine sample (n = 123; 92%; 95% CI: 84–97) and the cervicovaginal self-sample (n = 108; 81%; 95% CI: 70–88). Trust in correct collection of the cervicovaginal self-sample was significantly lower compared to collection of the urine sample (*p* < 0.001). Furthermore, the majority of the women were somewhat confident or confident that both sampling methods resulted in reliable test-outcomes: 121 (90%; 95% CI: 81–95) for urine collection versus 107 (80%; 95% CI: 70–88) for cervicovaginal self-sampling. Overall, women were more confident in the test-results of urine collection than cervicovaginal self-sampling (*p* < 0.001). Cervicovaginal self-sampling was perceived as painful in eight women (6%; 95% CI: 3–14).

### 3.5 Sampling preference for future cervical cancer screening

The majority of the women preferred urine collection (n = 39; 28%; 95% CI: 19–39) for future cervical cancer screening. Cervicovaginal self-sampling was preferred by 19 women (14%; 95% CI: 8–23). In addition, 13 women (9%; 95% CI: 5–18) preferred cervicovaginal self-sampling and urine collection equally, and clinician-taken cervical scrapes were preferred by 32 patients (23%; 95% CI: 15–34). Five women (4%; 95% CI: 1–11) preferred urine collection and clinician-taken cervical scrapes equally. Thirty women (21%; 95% CI: 14–32) did not have a sampling preference, and data was missing in two women (1%; 95% CI: 0–7) (Supplemental Table 2).

When the preferences for future cervical cancer screening are compared between women with a history of attending cervical cancer screening (n = 104) versus the women with no history of attending cervical cancer screening (n = 36), preferences for sampling differed (*p* = 0.004). Women with a history of attending cervical cancer screening, tended to prefer clinician-taken cervical scrapes (n = 30, 29%; 95% CI: 19–42) more frequently than women with no history of attending cervical cancer screening (n = 2, 6%; 95% CI: 1–25). In contrast, women with no history of attending cervical cancer screening tended to prefer urine collection (n = 14, 39%; 95% CI: 21–61) more frequently than women with a history of attending cervical cancer screening (n = 25, 24%; 95% CI: 15–37).

## 4 Discussion

This study shows that urine samples and cervicovaginal self-samples are considered easy to collect in a home-based setting. The majority of the women evaluated sampling instructions as clear, had trust they correctly executed both sampling methods, and expected the obtained results to be reliable. Urine collection was found to be more reliable and acceptable, compared to cervicovaginal self-sampling. The majority of the women preferred urine collection as sampling method in future cervical cancer screening.

As mentioned previously, urine collection has been reported as the most preferred sampling method for cervical cancer screening by others as well (Leeman et al., 2017, Ørnskov et al., 2021, Rohner et al., 2020, Sargent et al., 2019, Tranberg et al., 2020). These studies also showed that women were more confident in collecting a urine sample compared to a cervicovaginal self-sample (Sargent et al., 2019, Tranberg et al., 2020). The rating of the second-best preferred screening method, after urine collection, differs between studies: Leeman et al., 2017, Sargent et al., 2019, and Rohner et al. (2020) showed a preference for cervicovaginal self-sampling over clinician-taken samples, while patients in the study of Tranberg et al. (2020) and this study favoured clinician-taken samples over cervicovaginal self-sampling. A possible explanation why women in this study favor clinician-taken cervical scrapes over cervicovaginal self-samples, is their familiarity with collecting a clinician-taken cervical scrape and the fact that the majority of women in our study had a history of regular attendance in cervical cancer screening. It is expected that these results do not apply for non-attenders. This is supported by our findings, which showed a tendency in women who had a history of attending cervical cancer screening to prefer clinician-taken cervical scrapes (n = 30, 29%; 95% CI: 19–42) more frequently than women with no history of attending cervical cancer screening (n = 2, 6%; 95% CI: 1–25).

In general, cervicovaginal self-sampling is known to be preferred over clinician-taken sampling (Polman et al., 2019), and might lead to higher screening attendance rates (Gök et al., 2010, Polman et al., 2019, Verdoodt et al., 2015). In particular young women could have the greatest benefit of self-sampling, as their attendance rates in regular cervical cancer screening are lower (Albrow et al., 2014, Peto et al., 2004). However, in the Netherlands, the overall attendance rates within cervical cancer screening have decreased from 64 − 66% in 2012–2015 to 50% in 2020, despite of the introduction of cervicovaginal self-sampling, which has been offered as an option since 2017 (Aitken et al., 2021, Integraal Kankercentrum Nederland, 2021, Erasmus Medisch Centrum and Pathologisch-Anatomisch Landelijk Geautomatiseerd Archief, 2017). In total, only 5–8% of attendees choose to participate in cervical cancer screening by cervicovaginal self-sampling (Integraal Kankercentrum Nederland, 2021).

In order to increase the attendance rates, the Dutch Health Council recently advised to send self-sampling kits to all women invited for cervical screening, since this opt-out approach is considered to be more effective compared to the currently used opt-in approach (Arbyn et al., 2018, Dutch Health Council, 2021). However, this effect might be limited in women with a lower socio-economic status, women with a migration background, or women who have not attended cervical cancer screening for over ten years, because they are less prone to accept cervicovaginal self-sampling than the general population (Harder et al., 2018). Furthermore, less trust and low self-efficacy expectations towards performing correct cervicovaginal self-sampling might explain why only 5–8% of women attending cervical cancer screening participate by the option of cervicovaginal self-sampling (Integraal Kankercentrum Nederland, 2021, Polman et al., 2019, Williams et al., 2017). The evident preference for urine collection, reported by us and others as mentioned above, strengthens the expectation that non-attendees in cervical cancer screening will be responsive to collect a urine sample (Ducancelle et al., 2015, Lefeuvre et al., 2020).

A major strength of this study is that the urine and cervicovaginal self-samples were collected in a home-based setting without supervision from health care professionals. Therefore, it supports the further investigation of the implementation of urine collection as cervical cancer screening method. Another strength of this study is the high response rate (97%) and that urine was collected in basic urine collection cups, which are affordable (Hernandez-Lopez et al., 2021). The main limitation of this study is that the majority of the women had a history of attending cervical cancer screening (n = 104, 74%) and that all women had an abnormal cervical cytology prior to study participation. Hence, this study population is not the underscreened population that urine collection would provide a solution for. Also, a limitation of this study is the narrow scope of focus of the questionnaire and the limited number of open-ended responses. Future studies with validated and expanted questionnaires among non-attenders are needed. Finally, a limited number of women did not answer the questions about urine collection and cervicovaginal self-sampling. This suggests that in some cases the questionnaire was completed before urine collection and cervivovaginal self-sampling, and may have resulted in misundestanding and a bias towards a higher preference for clinician-taken sampling.

In conclusion, urine is easy to collect in a home-based setting and women have trust in the reliability of the corresponding results. In this study population, urine collection is the most preferred method for future cervical cancer screening, over collecting a clinician-taken cervical scrape or a cervicovaginal self-sample. Given the high acceptability, introducing urine collection in cervical cancer screening has the potential to increase screening participation and thus improve cervical cancer prevention.

## Funding

This research was funded by the Hanarth Foundation, the Weijerhorst Foundation and the KWF Dutch Cancer Society. The funders had no role in the design of the study; in the collection, analysis or interpretation of data; in the writing of the manuscript; or in the decision to publish the results.

### CRediT authorship contribution statement

**Mirte Schaafsma:** Investigation, Formal analysis, Writing – original draft. **Rianne van den Helder:** Investigation, Formal analysis, Writing – original draft. **Maaike C.G. Bleeker:** Conceptualization, Writing – review & editing. **Fleur Rosier-van Dunné:** Writing – review & editing. **Irene A.M. van der Avoort:** Writing – review & editing. **Renske D.M. Steenbergen:** Conceptualization, Funding acquisition, Writing – review & editing. **Nienke E. van Trommel:** Conceptualization, Funding acquisition, Writing – review & editing, Supervision.

## Conflicts of interest

The authors declare the following financial interests/personal relationships which may be considered as potential competing interests:

R.D.M.S. has a minority share in Self-screen B.V., a spin-off company of Amsterdam UMC, location VUmc. The remaining authors declare that they have no known competing financial interests or personal relationships that could have appeared to influence the work reported in this paper.
