# Supplementary material for: Experiences and preferences towards collecting a urine and cervicovaginal self-sample among women attending a colposcopy clinic
Source: Prev Med Rep. 2022 Feb 28;26:101749. doi: 10.1016/j.pmedr.2022.101749 (PMC8897716; doi:10.1016/j.pmedr.2022.101749)
Supplement: Supplemental Tables 1 and 2 [file mmc1.docx]

|  |  |  |  |  |  |  |  | **1: Extremely negative** | | **2: Negative** | | **3: Neutral** | | **4: Positive** | | **5: Extremely positive** | | **Total**  **number of respondents** | | **Missing** | | **Total** |
| --- | --- | --- | --- | --- | --- | --- | --- | --- | --- | --- | --- | --- | --- | --- | --- | --- | --- | --- | --- | --- | --- | --- |
| **Questions regarding cervical scrapes** | **Questions** | **Sample type** | **Mean** | **95% CI** | **Median** | **Range** | **P-value** | **n** | **%** | **n** | **%** | **n** | **%** | **n** | **%** | **n** | **%** | **n** | **%** | **n** | **%** | **n** |
|  | **Comfortable** | Cervical scrape | 4.2 | 4.0-4.4 | 5 | 1-5 | NA | 6 | 4% | 6 | 4% | 23 | 16% | 23 | 16% | 81 | 58% | 139 | 99% | 1 | 1% | 140 |
|  | **Painful** | Cervical scrape | 2.3 | 2.1-2.5 | 2 | 1-5 | NA | 61 | 44% | 17 | 12% | 25 | 18% | 27 | 19% | 8 | 6% | 138 | 99% | 2 | 1% | 140 |
|  | **Trust in correct sampling by clinician** | Cervical scrape | 4.7 | 4.6-4.8 | 5 | 1-5 | NA | 1 | 1% | 1 | 1% | 11 | 8% | 12 | 9% | 112 | 80% | 137 | 98% | 3 | 2% | 140 |
| **Questions regarding urine collection and cervicovaginal self-samples** | **Question** | **Sample type** | **Mean** | **95% CI** | **Median** | **Range** | **P-value** | **n** | **%** | **n** | **%** | **n** | **%** | **n** | **%** | **n** | **%** | **n** | **%** | **n** | **%** | **n** |
|  | **Clear instructions** | Urine collection | 4.8 | 4.7-4.9 | 5 | 3-5 | <0.001 | 0 | 0% | 0 | 0% | 6 | 4% | 11 | 8% | 110 | 79% | 127 | 91% | 13 | 9% | 140 |
|  |  | Cervicovaginal self-sampling | 4.6 | 4.5-4.8 | 5 | 1-5 |  | 1 | 1% | 2 | 1% | 8 | 6% | 22 | 16% | 98 | 70% | 131 | 94% | 9 | 6% | 140 |
|  | **Acceptable experience** | Urine collection | 4.6 | 4.5-4.7 | 5 | 3-5 | <0.001 | 0 | 0% | 0 | 0% | 12 | 9% | 31 | 21% | 89 | 64% | 132 | 94% | 8 | 6% | 140 |
|  |  | Cervicovaginal self-sampling | 4.3 | 4.1-4.4 | 5 | 1-5 |  | 1 | 1% | 3 | 2% | 21 | 15% | 41 | 29% | 67 | 48% | 133 | 95% | 7 | 5% | 140 |
|  | **Trust in correct sampling** | Urine collection | 4.6 | 4.5-4.7 | 5 | 2-5 | <0.001 | 0 | 0% | 2 | 1% | 8 | 6% | 31 | 22% | 92 | 66% | 133 | 95% | 7 | 5% | 140 |
|  |  | Cervicovaginal self-sampling | 4.1 | 3.9-4.2 | 4 | 1-5 |  | 2 | 1% | 7 | 5% | 17 | 12% | 59 | 42% | 49 | 35% | 134 | 96% | 6 | 4% | 140 |
|  | **Trust in reliable test-results** | Urine collection | 4.5 | 4.4-4.6 | 5 | 2-5 | <0.001 | 0 | 0% | 2 | 1% | 11 | 8% | 38 | 27% | 83 | 59% | 134 | 96% | 6 | 4% | 140 |
|  |  | Cervicovaginal self-sampling | 4.1 | 4.0-4.3 | 4 | 1-5 |  | 1 | 1% | 8 | 6% | 17 | 12% | 55 | 39% | 52 | 37% | 133 | 95% | 7 | 5% | 140 |
|  | **Pain experience** | Cervicovaginal self-sampling | 1.4 | 1.3-1.6 | 1 | 1-4 | <0.001 | 99 | 71% | 13 | 9% | 10 | 7% | 8 | 6% | 0 | 0% | 130 | 93% | 10 | 7% | 140 |

**Supplemental Table 1.** Outcomes of the questionnaires (n = 140), percentages include missing responses of women. Questions were rated from extremely negative to extremely positive. E.g. for clear instructions, extremely positive (or 5) indicates that the respondent found the instructions to be very clear and an extremely negative (or 1) indicates that the respondent found the instructions very unclear. Important to notice: pain experience was rated from extremely negative (or 1) indicating no painful experience to extremely positive (or 5) indicating a very painful experience. Abbreviations: CI: Confidence interval.

| **Preferences** | **History of attending cervical cancer screening at least once (n = 104)** | | **No history of attending cervical cancer screening (n = 36)** | | **Total**  **(n = 140)** | |
| --- | --- | --- | --- | --- | --- | --- |
|  | **n (%)** | **95% CI** | **n (%)** | **95% CI** | **n (%)** | **95% CI** |
| Clinician-taken cervical scrape (%) | 30 (29) | 19-42 | 2 (6) | 1-25 | 32 (23) | 15-34 |
| Urine collection (%) | 25 (24) | 15-37 | 14 (39) | 21-61 | 39 (28) | 19-39 |
| Cervicovaginal self-sampling (%) | 17 (16) | 9-28 | 2 (6) | 1-25 | 19 (14) | 8-23 |
| Equal preference for clinician-taken cervical scrape, urine collection, and cervicovaginal self-sampling, (%) | 17 (16) | 9-28 | 13 (36) | 19-58 | 30 (21) | 14-32 |
| Equal preference for cervicovaginal self-sampling or urine collection (%) | 8 (8) | 3-18 | 5 (14) | 5-35 | 13 (9) | 5-18 |
| Equal preference for clinician-taken cervical scrape or urine collection (%) | 5 (5) | 2-14 | 0 ( 0) | 0-17 | 5 (4) | 1-11 |
| Missing (%) | 2 (2) | 0-10 | 0 ( 0) | 0-17 | 2 (1) | 0-7 |

**Supplemental Table 2:** Preferences for sample collection in future cervical cancer screening in all participating women (n = 140) and subgroups based on history of attending cervical cancer screening. In the group with a history of attending cervical cancer screening, 19 women also had a history of opportunistic cervical screening in the context of complaints. In the group of women with no history of attending cervical cancer screening, women were included with a history of opportunistic cervical screening or in the context of complaints, and one woman with no history of cervical screening.
