# Supplementary material for: Experiences and preferences towards collecting a urine and cervicovaginal self-sample among women attending a colposcopy clinic
Source: Prev Med Rep. 2022 Feb 28;26:101749. doi: 10.1016/j.pmedr.2022.101749 (PMC8897716; doi:10.1016/j.pmedr.2022.101749)
Supplement: Supplement 1 [file mmc2.docx]

Questionnaire SOLUTION 2 -study

After you have collected both study samples (urine and cervicovaginal self-sample), we would like to ask you to complete this questionnaire.

**Please check the box or fill in the answer which applies to you.**

1. What is your age? year
2. What is the highest level of education you have completed?

□ No / Primary school

□ Secondary education: MAVO / VMBO / LBO (Dutch educational levels)

□ Secondary education: HAVO / VWO (Dutch educational levels)

□ Secondary vocational education: MBO (Dutch educational level)

□ Higher professional education: HBO (Dutch educational level)

□ University: WO

**The next questions are about your experience on having a cervical scrape taken.**

1. Have you ever had a cervical scrape taken?

□ No, I have never had a cervical scrape taken

□ Yes, in the context of cervical cancer screening

□ Yes, in the context of complaints

1. When was the last time you had a cervical scrape taken?

□ 0 – 6 years ago

□ 7 – 12 years ago

□ 13 years or longer ago

1. How is the current abnormality of the cervix discovered?

□ by participating in the cervical cancer screening program, for which a cervical scrape was taken by the general practitioner/assistant

□ by participating in the cervical cancer screening program, first by collecting a cervicovaginal self-sample, afterwards a cervical scrape was taken by the general practitioner

□ a cervical scrape was taken by the general practitioner/assistant because of complaints

□ other: …….

1. In order to respond on the question how you experienced the cervical scrape taken by the general practitioner/assistant, we would like to ask you to put a cross in the box corresponding to the answer that is most applicable to you. In total you will put 3 crosses for this question.

| 1 | 2 | 3 | 4 | 5 |  |
| --- | --- | --- | --- | --- | --- |
| Disagree | Partly disagree | Neutral | Partly agree | Agree |  |
| I felt comfortable |  |  |  |  |  |
| I experienced it as painful |  |  |  |  |  |
| I trust that the smear was taken properly. |  |  |  |  |  |

**The next questions are related to your experiences with the cervicovaginal self-sample (method 1) and the collection of urine (method 2). For both methods we would like to ask you to put a cross in the box corresponding to the answer that is most applicable to you.** In total you will put 2 crosses for each question.

1. How did you experience the instructions for urine collection and cervicovaginal self-sampling?

| Not clear |  | | | | Clear |  |
| --- | --- | --- | --- | --- | --- | --- |
| 1 | 2 | 3 | 4 | | 5 |  |
| **Cervicovaginal self-sampling** |  |  |  | |  |  |
| **Urine** |  |  |  | |  |  |

1. How did you experience both collection methods?

| Not acceptable |  | | | | Acceptable |  |
| --- | --- | --- | --- | --- | --- | --- |
| 1 | 2 | 3 | 4 | | 5 |  |
| **Cervicovaginal self-sampling** |  |  |  | |  |  |
| **Urine** |  |  |  | |  |  |

1. Do you trust you correctly self-collected both samples?

| Unconfident |  | | | | Confident |  |
| --- | --- | --- | --- | --- | --- | --- |
| 1 | 2 | 3 | 4 | | 5 |  |
| **Cervicovaginal self-sampling** |  |  |  | |  |  |
| **Urine** |  |  |  | |  |  |

1. Do you trust in reliable test-results of both sampling methods?

| Unconfident |  | | | | Confident |  |
| --- | --- | --- | --- | --- | --- | --- |
| 1 | 2 | 3 | 4 | | 5 |  |
| **Cervicovaginal self-sampling** |  |  |  | |  |  |
| **Urine** |  |  |  | |  |  |

**The next question is related to the cervicovaginal self-sampling.**

1. Did you experience pain collecting the cervicovaginal self-sample?

| No pain |  | | | | Very painful |  |
| --- | --- | --- | --- | --- | --- | --- |
| 1 | 2 | 3 | 4 | | 5 |  |
| **Cervicovaginal self-sampling** |  |  |  | |  |  |

**The next question is related to your sampling preference for future cervical cancer screening.**

1. If you are allowed to choose the screening method, which method would you prefer?

□ Cervicovaginal self-sampling

□ Collection of urine

□ Cervical scrape taken by general practitioner/assistant

□ I have no preference

***In the box below it is possible to give extra information about your answers:***

**This is the end of the questionnaire.**

Could you check if you have responded to **all** questions?

**Thank you for filling in the questionnaire!**
